# Unified deep learning models for enhanced lung cancer prediction with ResNet-50–101 and EfficientNet-B3 using DICOM images

**DOI:** 10.1186/s12880-024-01241-4

**Published:** 2024-03-18

**Authors:** Vinod Kumar, Chander Prabha, Preeti Sharma, Nitin Mittal, S. S. Askar, Mohamed Abouhawwash

**Affiliations:** 1 Department of Computer Science and Engineering, Chandigarh University, Mohali, Punjab India; 2 Chitkara University Institute of Engineering and Technology, Chitkara University, Punjab, India; 3 Skill Faculty of Engineering and Technology, Shri Vishwakarma Skill University, Palwal, Haryana India; 4 Department of Statistics and Operations Research, College of Science, King Saud University, P.O. Box 2455, 11451 Riyadh, Saudi Arabia; 5 Department of Mathematics, Faculty of Science, Mansoura University, Mansoura, 35516 Egypt

**Keywords:** Lung Cancer, Deep Learning, Cancer Detection, EfficientNet-B3, ResNet-50, ResNet-101, Fusion

## Abstract

Significant advancements in machine learning algorithms have the potential to aid in the early detection and prevention of cancer, a devastating disease. However, traditional research methods face obstacles, and the amount of cancer-related information is rapidly expanding. The authors have developed a helpful support system using three distinct deep-learning models, ResNet-50, EfficientNet-B3, and ResNet-101, along with transfer learning, to predict lung cancer, thereby contributing to health and reducing the mortality rate associated with this condition. This offer aims to address the issue effectively. Using a dataset of 1,000 DICOM lung cancer images from the LIDC-IDRI repository, each image is classified into four different categories. Although deep learning is still making progress in its ability to analyze and understand cancer data, this research marks a significant step forward in the fight against cancer, promoting better health outcomes and potentially lowering the mortality rate. The Fusion Model, like all other models, achieved 100% precision in classifying Squamous Cells. The Fusion Model and ResNet-50 achieved a precision of 90%, closely followed by EfficientNet-B3 and ResNet-101 with slightly lower precision. To prevent overfitting and improve data collection and planning, the authors implemented a data extension strategy. The relationship between acquiring knowledge and reaching specific scores was also connected to advancing and addressing the issue of imprecise accuracy, ultimately contributing to advancements in health and a reduction in the mortality rate associated with lung cancer.

## Introduction

Human bodies are composed of different cells. Beating cancer happens when one of these cells encounters wild and unordinary progression due to cellular changes [1]. The World Prosperity Organization reports that cancer is the diminutive driving cause of passing around the world. The recurrence of as of late analyzed cancer cases continues to rise each year [2] and [3]. The mortality rate for cancer is 6.28% for females and 7.34% for folks. Lung and verbal cancer in men reports 25% of cancer-connected passings, whereas breast and verbal cancer pitch in 25% of female cancer-linked passings. The cancer estimations are routinely changed and wrap data from [4–7]. Table 1 shows the rates meaning the fundamental components behind cancer.

**Table 1:**
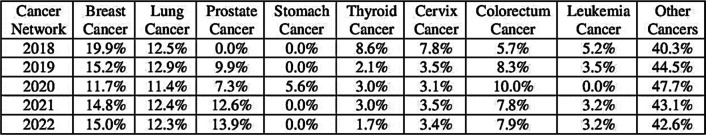
Cancer statistics: A global comparison (India 2018 vs. World 2020)

**Fig. 1:**
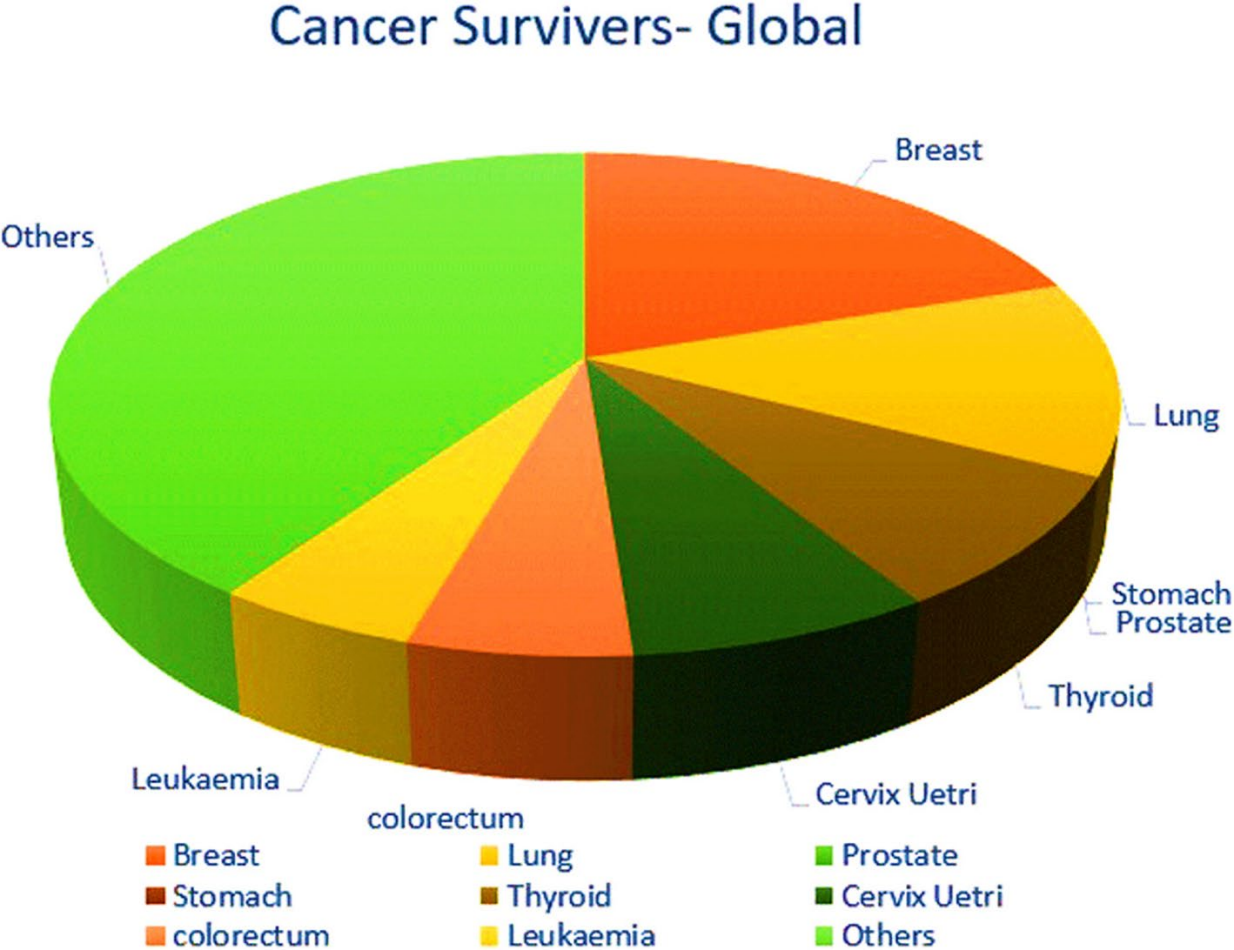
Trends in Cancer Survivorship in India and Globally

In 2020, lung cancer has risen as the first dangerous outline of cancer, point by point [8] and [9]. The ensuing three exceedingly deadly malignancies were breast, liver, and stomach cancer, bookkeeping for 6.9%, 8.3%, and 7.7% of cancer-related passings, individually. Figure 1 outlines the worldwide measurements of cancer mortality up to 2020.

The Cancer Actualities and Figure think gauges that the year 2022 will witness an amazing 1,918,030 modern cases of cancer. Over about nine decades, from 1930 to 2019, lung cancer has reliably risen as the essential cause of passing among guys, as uncovered by the study's discoveries. Among the differing clusters of cancer sorts, stomach, colon, and prostate cancers win as the foremost predominant among guys. Shockingly, despite lower and larger cancer rates, lung cancer proceeds to claim the lives of more females than any other frame of cancer. On the other hand, breast, stomach, and colon cancers rule the scene of cancer among ladies [10]. In the afterward times, the field of restorative ponders has seen an earth-shattering development inside the abuse of fake insights and machine learning techniques [11, 12]. These cutting-edge methodologies have been instrumental in the improvement of prescient models for different infections, including cancer. Eminently, the professional deep-learning models [13] to estimate lung cancer stands as a groundbreaking restorative apparatus at this dynamic time.

The proposed device utilizes profoundly proficient deep-learning models to classify major lung cancer. In organizing to progress the precision of display lung cancer anticipation structure, the proposition for ensemble and fusion strategies are provided.

A novel part of the current study is the development of a support system using three different deep-learning models (ResNet-50, EfficientNet-B3, and ResNet-101) combined with transfer learning thereby reducing the related mortality rate and improving health.

The purpose of the study is to provide further knowledge that the usage of deep learning techniques improves cancer research. Specifically, the authors discuss how deep learning models can be applied to medical research and diagnostics to improve health outcomes and reduce mortality rates and how ensemble learning enhances lung cancer prediction.

### Contributions

- To detect lung cancer subtypes, a support system was developed by combining transfer learning with three different deep learning models (ResNet-50, EfficientNet-B3, and ResNet-101).
- A considerable level of accuracy was attained in the classification of Squamous cells with the use of Fusion Models and ResNet-50.
- Implement a data expansion strategy to prevent overfitting and enhance data collection and planning.
- Using ensemble and fusion techniques, lung cancer precision has improved, which might lead to better health outcomes and potentially a decrease in the mortality rate from the disease.

### Motivation

- To prevent lung cancer, research is being conducted to create deep learning models.
- To significantly improve health by improving the accuracy of diagnosis and lowering the disease's death rate.

### Structure of paper

The overall organization starts with a thorough introduction and discusses the current state of cancer survivorship in India and throughout the world. A summary of deep learning systems in therapeutic applications and the parallels between deep learning models and cancer diagnosis are also provided. The related research Sect. 2nd expands the scope of in-depth learning about cancer research, supported by perceptible trends in mortality from 2012 to 2023. The materials and methods Sect. 3rd outlines the techniques used, which include the use of convolutional neural networks (CNNs) and a transfer learning model that consists of ResNet50, ResNet101, and EfficientNet-B3. These are complemented by images that illustrate the power of transfer learning in deep networks. Section 4, data section provides a detailed evaluation of the combined LIDC-IDRI, displaying several types of lung cancer images. To enhance lung cancer detection, the results and discussion section include data from the fusion of three deep learning models. In Sect. 5, an in-depth review of the training and verification procedures is provided by the experimental analysis section. This understanding is provided via visual representations of the accuracy and loss of training and verification curves, confusion matrices, and comparisons of deep learning models for cancer detection performance. Hence, this well-planned organization of the manuscript ensures clarity, coherence, and thoroughness in the methodology, results, and research discussion presentations. Finally, Sect. 6 concludes the findings and scope for future work.

### Related work

Over the past decade, the collection of multimodality information has driven a noteworthy increment within the utilization of information examination in well-being data frameworks. The therapeutic field has experienced fast development with the advancement of machine learning models to oversee and examine this endless sum of restorative information, as referenced in [14]. Deep Learning, which is based on fake neural systems, has developed as an advanced machine learning strategy with the potential to convert the counterfeit insights industry, as famous in [15].

DL has demonstrated its value in the medical sector by effectively managing previously challenging tasks. It offers an extent of organized sorts with different capabilities, empowering the proficient to take care of expansive volumes of restorative information, including literary data, sound signals, restorative images, and recordings. These DL systems, moreover, known as models, have been demonstrated to be profoundly viable apparatuses in various restorative frameworks [16–19]. Both ML and DL models have achieved success in various medical domains, including cancer prevention, detection, and COVID-19 diagnosis [20–22] and medical data analysis. DL models play a prominent role in medicine, with the selection and configuration of networks depending on the specific field, data volume, and research objectives. For a comprehensive list of commonly utilized DL networks and their distinctive features in the medical industry [23, 24], refer to Table 2.

**Table 2:** Deep learning systems in therapeutic applications

Network Types	Key Characteristics	Detailed Description	Notable Remarks
Deep auto-encoder [25]	The input–output layers of the framework have a break indeed with the number of neurons, which must be at least 2	Utilized for unsupervised learning, connected for dimensionality decrease or change	Utilized for include extraction and determination
Deep Boltzmann Machine [26]	The layers in the address are undirected and have a measurement of 2 or more. These layers can be categorized as obvious or covered up, with no nearness of input or yield layers	The undirected associations encourage both administered and unsupervised learning, whereas too minimizing the time required for the learning handle	Not appropriate for huge datasets
Convolutional Neural Networks [27]	Incorporates classification, convolution, pooling, and completely connected layers. Utilize an enactment work that's not straight. Acknowledges input specifically as an image	Utilized to fathom therapeutic image categorization issues for unremitting illness and cancer discovery	Apply the network's highlight extraction preparation. Not each neuron is wired together. takes too much data to memorize
ResNet-50 [28]	A 50-layer deep CNN sort that's more advanced comprises leftover units that skip associations	Utilized to classify therapeutic images with moved forward execution Requires more preparing time than CNN but performs impressively superior	It takes much data as well to memorize
GoogLeNet [29]	Inception CNN: concurrent convolution with diverse part sizes, high-performance therapeutic image classification	In Initiation, CNN trains sped up more than ResNet-50, even though its execution was a little better	Request a gigantic set of data to memorize effectively
EfficientNet [30]	CNNs can make strides by expanding their profundity, width, and determination	Utilized to fathom a part of the image categorization issue. Compared to ResNet-50 and 101	It is smaller and speedier

Machine learning and deep learning are progressively being utilized in therapeutic investigation, and cancer avoidance and discovery could be key regions of the center [31]. This article surveys the latest things in this field, highlighting the foremost promising progress.

**Fig. 2:**
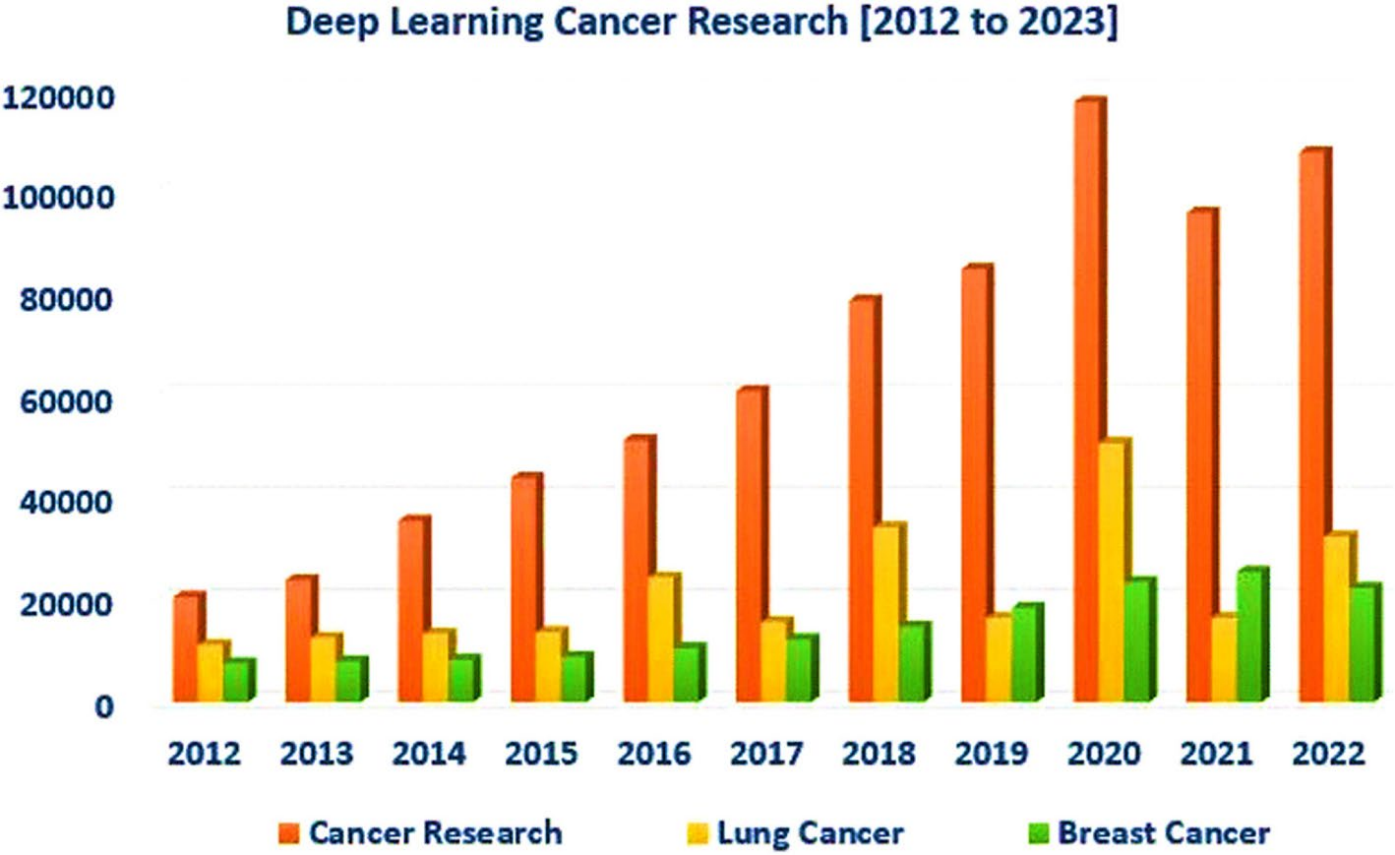
Trends in deep learning cancer research mortality, 2012–2023

A look at "Google Researcher" gives important insights into cancer investigations from 2014 to 2022. The information uncovered in Fig. 2, highlights the expanding intrigue in utilizing deep learning in cancer investigations. Additionally, it illustrates that lung cancer receives more focus compared to breast cancer. The study indicates that the breast and lung cancer ratios are the highest. These facts were gathered by Google Scholar on October 24 at noon.

Table 3 illustrates that previous research had deficiencies, with some studies exhibiting poor accuracy due to the use of incorrect methodologies or parameters. Certain investigations employed sophisticated models, but most of the research only utilized one or two indicators, which is inadequate for evaluating accuracy and effectiveness. To attain tall execution with a low-computational show, the show ponder will consider the preferences of outfit learning, exchange learning, and particular profound models with moo computational effectiveness.

**Table 3:** Similarity of deep learning models for cancer determining

Researcher	Area characterization	DL illustrate	Information set	Outcome
[26]	Advanced Breast Tomosynthesis vs. Computerized Mammography	Pretrained VGG16	Breast Screen Norway screening program	The rate of breast cancer detected by screening is comparable between computerized breast tomosynthesis and stepwise mammography in a population-based screening program
[27]	Precise aspiratory nodule discovery	Convolutional Neural Systems (CNNs)	LIDC-IDRI dataset	Affectability of 92.7% with 1 untrue positive per filter and affectability of 94.2% with 2 wrong positives per check for lung knob discovery on 888 checks. Utilization of thick Most extreme Concentrated Projection (MIP) images makes a difference distinguish little aspiratory knobs (3 mm-10 mm) and diminishes wrong positives
[32]	Pathogenesis of Oral Cancer	Not applicable (no deep learning model mentioned)	Not applicable (no dataset mentioned)	Audit and talk of key atomic concepts and chosen biomarkers embroiled in verbal carcinogenesis, particularly in verbal squamous cell carcinoma, with a center on deregulation amid diverse stages of verbal cancer advancement and movement
[33]	Liquid Biopsies for BC	Not applicable	Meta-analysis of 69 studies	ctDNA mutation rates for TP53, PIK3CA, and ESR1: 38%, 27%, and 32% respectively
[34]	Assessment of smartphone-based Employing Visual Review of the Cervix with Acidic Corrosive in helpful settings	Not appropriate	Information collected from 4,247 patients who experienced cervical cancer screening in rustic Eswatini from September 1, 2016, to December 31, 2018	Introductory Using inspiration rate expanded from 16% to 25.1% standard preparing, at that point dropped to a normal of 9.7 term refresher preparing, expanded once more to a normal of 9.6 before the beginning of mentorship, and dropped to a normal of 8.3% in 2018
[35]	Healthcare and Deep Learning	Deep Learning (Artificial Neural Network)	Electronic Health Data—8000	Improved predictive performance and applications in various healthcare areas, Accuracy- 97.5%
[36]	Computer-Aided Diagnosis (CAD) in Gastric Cancer	Not specified in the provided text	Histopathological images of gastric cancer (GHIA)	Summarizes image preprocessing, feature extraction, segmentation, and classification techniques for future researchers
[37]	Tumor organization of non-small cell lung cancer (NSCLC) with detailed insights	Two-step deep learning shows autoencoder and CNN) for NSCLC arranging	Preparing (*n* = 90), Approval (*n* = 8), Test cohorts (*n* = 37, *n* = 26) from open space (CPTAC and TCGA)	CPTAC Test Cohort: Precision:0.8649 Affectability:0.8000 Specificity:0.9412 AUC:0.8206 TCGA Test Cohort: Exactness:0.8077 Affectability:0.7692 Specificity:0.8462 AUC:0.8343
[38]	Precise location and classification of breast cancer	Pa-DBN-BC (Deep Conviction Arrange)	The entire slide histopathology image dataset from four information cohorts	86% accuracy
[39]	Skin Cancer Diagnosis	U-Net and VGG19	ISIC 2016, ISIC 2017, ISIC 2018	Palatable comes about compared to state-of-the-art
[40]	Rectal Adenocarcinoma Survival Prediction	DeepSurv model (seven-layer neural network)	Patients with rectal adenocarcinoma from the Soothsayer database	C index: 0.824 (preparation cohort) and 0.821 (test cohort) Factors influencing survival: age, gender, marital status, tumor evaluation, surgical status, and chemotherapy status. High consistency between test and cohort predictions
[41]	Prostate Cancer Diagnosis and Gleason Grading	Deep Residual Convolutional Neural Network	85 prostate core biopsy specimens digitized and annotated	Coarse-level accuracy: 91.5%, Fine-level accuracy: 85.4%
[42]	Tree-based BrT Multiclassification Demonstrate for Breast Cancer	Outfit tree-based deep learning demonstrates	BreakHis dataset (pretraining), BCBH dataset	Classification accuracy of 87. 50% to 100% for the four subtypes of BrT The proposed show is beyond the state of the art
[43]	Breast Cancer (BC)	Transfer Learning (TL)	MIAS dataset	80–20 strategy: Precision: 98.96D44 Affectability: 97.83D44 Specificity: 99.13D44 Accuracy: 97.35D44F-score: 97.66D44 AUC: 0.995 tenfold cross-validation strategy: Exactness: 98.87D44 Affectability: 97.27D44 Specificity: 98.2D44 Accuracy: 98.84D44 F-score: 98.04D44 AUC: 0.993
[44]	Screening for breast cancer with mammography	Deep learning and convolutional neural systems	Different datasets in advanced mammography and advanced breast tomosynthesis	AI calculations appearing guarantee in review information sets, AUC 0.91, advance considers required for real-world screening effect
[45]	Breast Cancer Diagnosis	Statistical ML and Deep Learning	Various breast imaging datasets	Recommendations for future work Accuracy 97%
[46]	Dermoscopic Expert	Crossbreed Convolutional, Neural Organize (hybrid-CNN)	ISIC-2016, ISIC-2017, ISIC-2018 AUC of 0.96, 0.95, 0.97 Advanced	AUC by 10.0% and 2.0% for ISIC-2016 and ISIC-2017 datasets, 3.0% higher balanced precision for ISIC-2018 dataset
[47]	Breast Cancer Classification	ResNet-50 pre-trained model	Histopathological images from Jimma College Therapeutic Center, 'BreakHis,' and 'zendo' online datasets	96.75 accuracy for twofold classification, 96.7 accuracy for generous sub-type classification, 95.78 accuracy for threatening sub-type classification, and 93.86 accuracy for review recognizable proof
[48]	Cancer-Net SCa	Custom deep neural organize plans	Universal Skin Imaging Collaboration (ISIC)	Made strides in precision compared to ResNet-50, decreased complexity, solid skin cancer discovery execution, empowered open-source utilization and improvement
[49]	Automating Medical Diagnosis	Transfer Learning, Image Classification, Object Detection, Segmentation, Multi-task Learning	Medical image data, Skin lesion data, Pressure ulcer, Segmentation data,	Cervical cancer: Sensitivity + 5.4%, Skin lesion: Accuracy + 8.7%, Precision + 28.3%, Sensitivity + 39.7%, Pressure ulcer: Accuracy + 1.2%, IoU + 16.9%, Dice similarity + 3.5%
[50]	Symptomatic Precision of CNN for Gastric Cancer Anticipating Attack Profundity of Gastric Cancer	Convolutional Neural Network (CNN)	17 studies, 51,446 images, 174 videos, 5539 patients	Sensitivity: 89%, Specificity: 93%, LR + : 13.4, LR–: 11, AUC: 0.94 Sensitivity: 82%, Specificity: 90%, LR + : 8.4, LR–: 20, AUC: 0.90
[51]	Image Quality Control for Cervical Precancer Screening	Deep learning gathering system	87,420 images from 14,183 patients with numerous cervical cancers think about	Accomplished higher execution than standard approaches
[52]	Breast Cancer Determination Utilizing Deep Neural Systems	Convolutional Neural Systems (CNN)	Mammography and histopathologic images	Moved forward BC conclusion with DL, utilized open and private datasets, pre-processing procedures, neural arrange models, and distinguished inquire about challenges for future advancements
[53]	HPV Status Prediction in OPC, Survival Prediction in OPC	Ensemble Model	492 OPC Patient Database	AUC: 0.83, Accuracy: 78.7% AUC: 0.91, Accuracy: 87.7%
[54]	Pathology Detection Algorithm	YOLOv5 with an improved attention mechanism	Gastric cancer slice dataset	F1_score: 0.616, mAP: 0.611; Decision support for clinical judgment
[55]	Cervical Cancer (CC)	HSIC, RNN, LSTM, AFSA	Not mentioned	Risk scores for recurrence CC patients using the AFSA algorithm
[56]	Hepatocellular carcinoma (HCC)	Inception V3	Genomic Data Commons Databases H&E images	Matthews’s correlation coefficient, 96.0 accuracy for benign/malignant classification, and 89.6 accuracy for tumor separation. Anticipated ten most common changed qualities (CTNNB1, FMN2, TP53, ZFX4) with AUCs from 0.71 to 0.89

## Materials and methods

### Convolutional Neural Network (CNN)

The CNN, or deep neural arrangement, takes a 2D image as input and produces classes or lesson probabilities as the yield. It is utilized in areas like therapeutic conclusion, individual distinguishing proof, and image classification. The CNN's structure incorporates convolution layers, pooling layers, and a completely associated layer [57].

The convolutional layer applies the convolution method, where a bit of estimate K*K is convolved with an image of measurements M*N. The bit moves over the image, duplicating each pixel by its encompassing pixels and including the items together to grant the convolution's yield. This yield is called the actuation outline, and its measure changes based on the number of channels utilized.

**Fig. 3:**
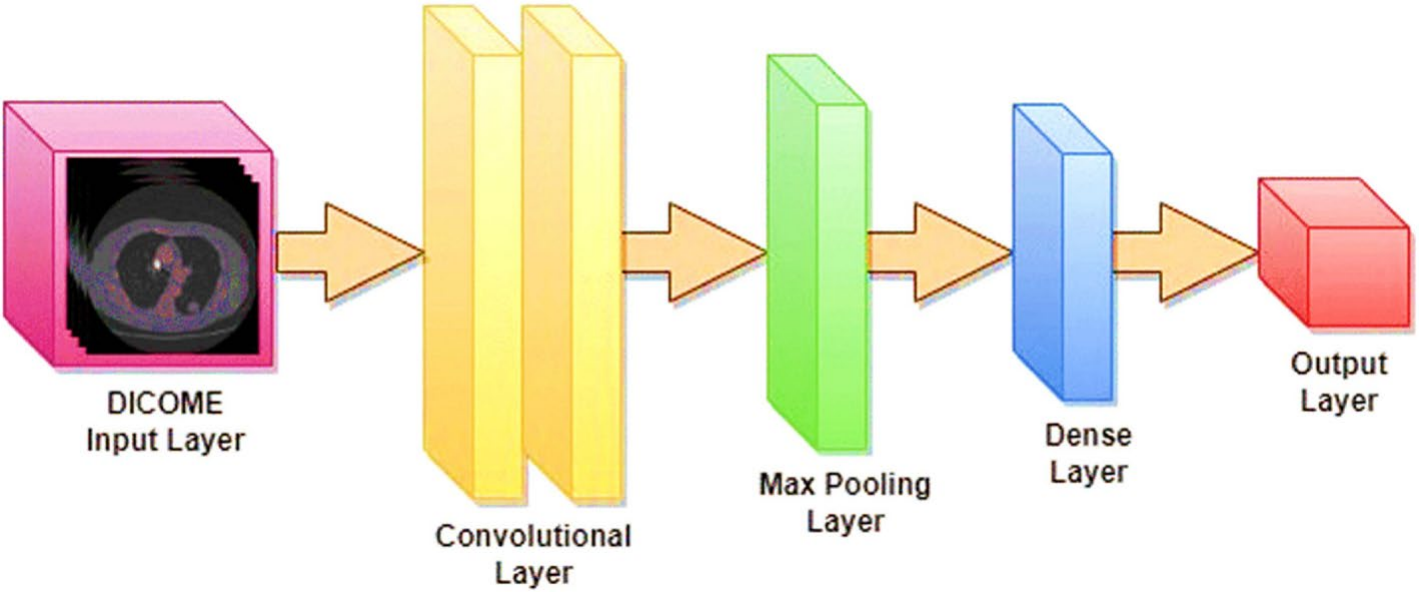
Building blocks of a convolutional neural network

The ultimate measure of the convolution is decided by components like walk (S) and cushioning (P). The walk shows the part measure, whereas cushioning includes columns and columns for border pixels. For example, a 5*5 part features a cushioning of 2. The yield measure is decided by the equation (W—F + 2P) / S + 1, where W is the image measure, F is the part measure, S is the walk, and P is the cushioning. The yield is at that point passed to a pooling layer, which diminishes the image. CNNs can utilize max pooling (selecting max esteem) or normal pooling (calculating normal). The components of a Convolutional Neural Network (CNN) are depicted in Fig. 3, which clarifies the complex architecture that is essential to deep learning for tasks involving classification and recognition of images.

All going-before layer neurons are associated with the Completely Associated Layer (FC). The neuron's esteem in this layer is decided by the whole of the weighted items of all past layer neurons. Non-linear actuation capacities like Sigmoid, Tanh, and ReLU are utilized in conventional CNN systems to evacuate boisterous pixels after convolution and pooling layers. These actuation capacities are connected recently to the pooling layer and after each convolutional layer. To form the ultimate convolution that comes about congruous with the FC layer, a straightening layer is commonly utilized.

### Proposed transfer learning models

**Fig. 4:**
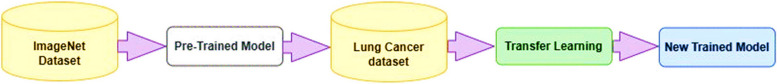
Visualizing the Power of Transfer Learning in Deep Networks

Three built-up models, to be specific ResNet-50, ResNet-101, and EfficientNet-B3, are utilized within the current ponder to examine the viability and execution of distinctive CNN engineering sorts. The concept of exchange learning, as delineated in Fig. 4, includes the utilization of pre-trained models for an unused problem that contrasts with the initial issue. The ResNet-50 and 101 [58] and EfficientNet-B3 [59] models have been already prepared to utilize the ImageNet dataset. In this examination, these models will be utilized to form expectations concerning lung cancer. The input image has three color channels and a standard pixel size of 224 by 224. The first convolutional layer of the ResNet architecture successfully extracts information from the input picture with a stride value of 2 by using a kernel size of 7 × 7, including 64 different kernels.

ResNet-50, a distinctive version of CNN, consolidates the remaining units created by [60]. ResNet-50 may be a 50-layer profound arrangement comprising one max pooling layer, one FC layer, and 48 convolutional layers. The essential advantage of ResNet-50 lies in its utilization of remaining units. These units successfully address the issue of vanishing angles experienced in prior profound systems. Within the ResNet-50 design, the remaining units are shown during each segment and serve as skipping associations, as delineated in Fig. 4.

As you dive advance into the profundities, the angle either vanishes or gets to be exceedingly minute. In any case, as you proceed to slip, the slope lessens. To check this, the ResNet design consolidates associations and leftover units that bypass different convolutional layers (three in ResNet-50), successfully anticipating the angle from reducing.

The design of ResNet-50 comprises 50 convolutional layers. The primary layer has 64 channels with a measure of 7*7 and a walk of 2. The ensuing max pooling layer (walk = 2) reduces the convolution estimate. This is often taken after by three convolution layers with 64 channels of estimate 1*1, 64 channels of measure 3*3, and 256 channels of measure 1*1. Three more convolution layers are taken after. The following four convolutional layers are composed of 512 estimated 1*1 channels, 128 estimated 3*3 channels, and 128 measure 1*1 channels. The other layer comprises 1024 channels of estimate 1*1, which is rehashed six times, alongside 256 channels of estimate 1*1, 256 channels of measure 3*3, and 256 channels of estimate 1*1.

The ResNet-50 network's last convolution layers include 2048 measured 1*1 channels, 512 estimated 3*3 channels, and 512 estimated 1*1 channels. The best layer of this organization, known as the FC layer or normal pooling layer, comprises 1000 tests speaking to the ultimate highlight vector. It utilizes a "Softmax" enactment work to classify images into different classes. In differentiation, RenNet101 utilizes the ImageNet dataset to prepare its 101 layers, joining an add-up to 44.5 million preparing parameters [61].

The authors presented EfficientNet [62], a CNN architecture that scales all measurements (profundity, breadth, and determination) through compound coefficients. They created a course of EfficientNet topologies that are both exact and compact, illustrating that it outperforms earlier models such as ResNet, Xception, NasNet, and Initiation in terms of computation. The network's three measurements are similarly scaled utilizing compound scaling, permitting the show to powerfully react to the input estimate.

### Dataset

Three different folders were created from the dataset of Chest CT-Scan images: 70% were put aside for training, 20% for validation, and 10% were set aside for testing. There are 613 images in the training dataset, 315 in the validation dataset, and 72 in the testing dataset. Adenocarcinoma, Large Cell Carcinoma, Squamous Cell Carcinoma, and Normal CT Image were the four distinct categories into which the authors meticulously categorized a dataset of 1,000 DICOM lung cancer images from the LIDC-IDRI repository [63]. The data collection was divided into three categories: training (70%), validation (20%), and testing (10%). Specifically, it contains 613 images in the training dataset and 315 and 72 images in the validation and test datasets. To accurately classify these images, the researchers used a robust deep learning model that included ResNet-50, ResNet-101, and EfficientNet-B3, with an emphasis on improving the prediction accuracy of lung cancer subtypes. The Fusion Model categorized Squamous Cells with 100% accuracy, whereas ResNet-50, EfficientNet-B3, and ResNet-101 all had 90% accuracy, with EfficientNet-B3 and ResNet-101 having considerably lesser precision. It also used a data augmentation approach to improve the data's resilience and reduce overfitting. after closely examining our models' performance across 35 time periods. According to our research, ResNet-101 and EfficientNet-B3 outperform ResNet-50. The findings highlight the ability of deep learning algorithms to make more accurate lung cancer diagnoses, which might lead to improvements in medical care and perhaps lower death rates.

**Fig. 5:**
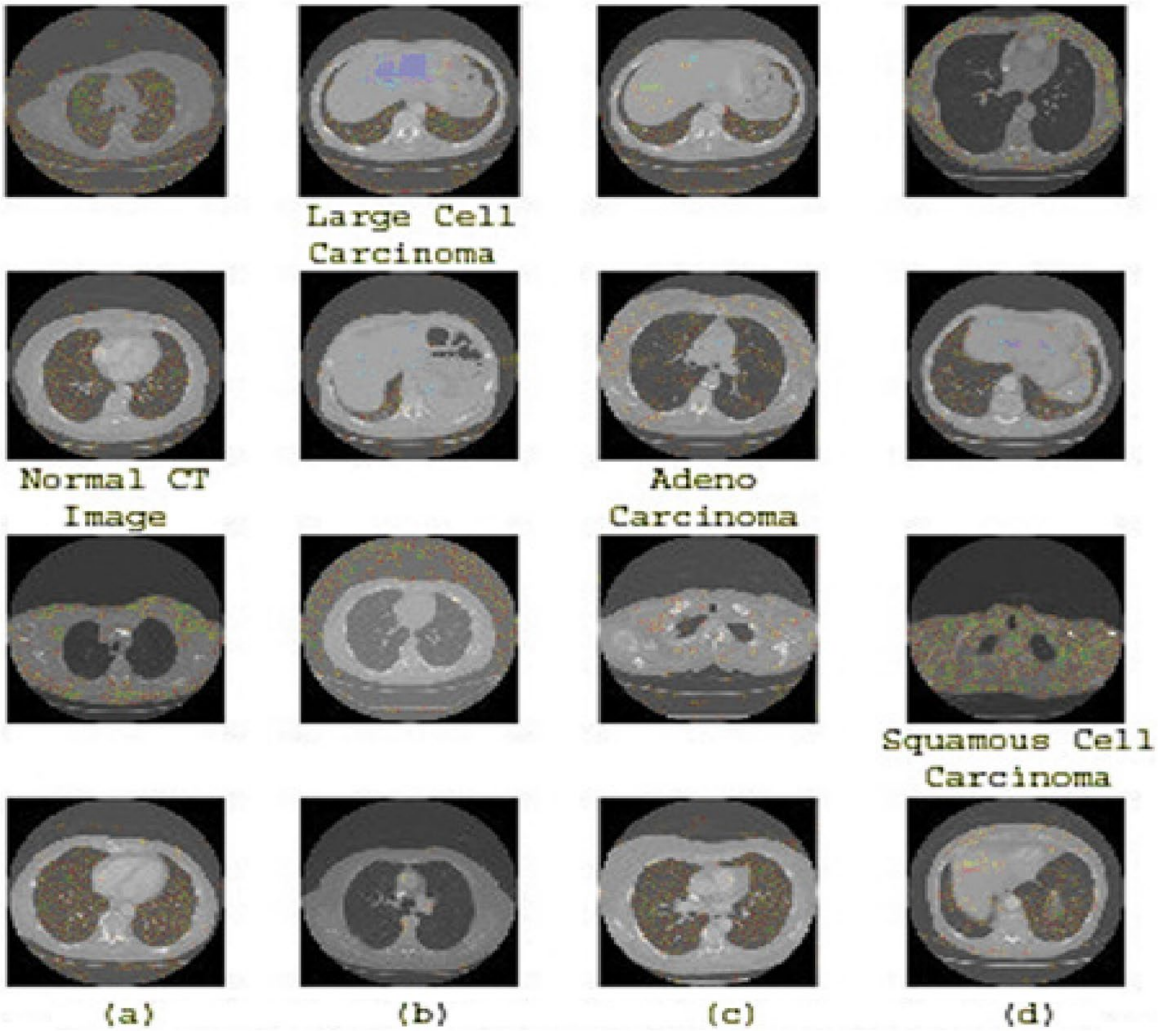
(**a**) Normal CT Image, **b** Large Cell Carcinoma, **c** Adenocarcinoma, **d** Squamous Cell Carcinoma

To distinguish between these kinds, the use of deep learning requires the use of a powerful classifier, as shown in Fig. 5 (a, b, c, d). This figure shows cases from different categories in the prepared data sets, highlighting the similarities between them, such as adenocarcinoma and large cells. The main challenge encountered in this data set lies in the similarities observed in the classifications. In any case, the application of the information expansion method will be used to address this concern considering the limited measurement of the dataset. Figure 5 (a, b, c, d) shows illustrations of the three forms of lung cancer as well as a healthy case.

## Results and discussion

**Fig. 6:**
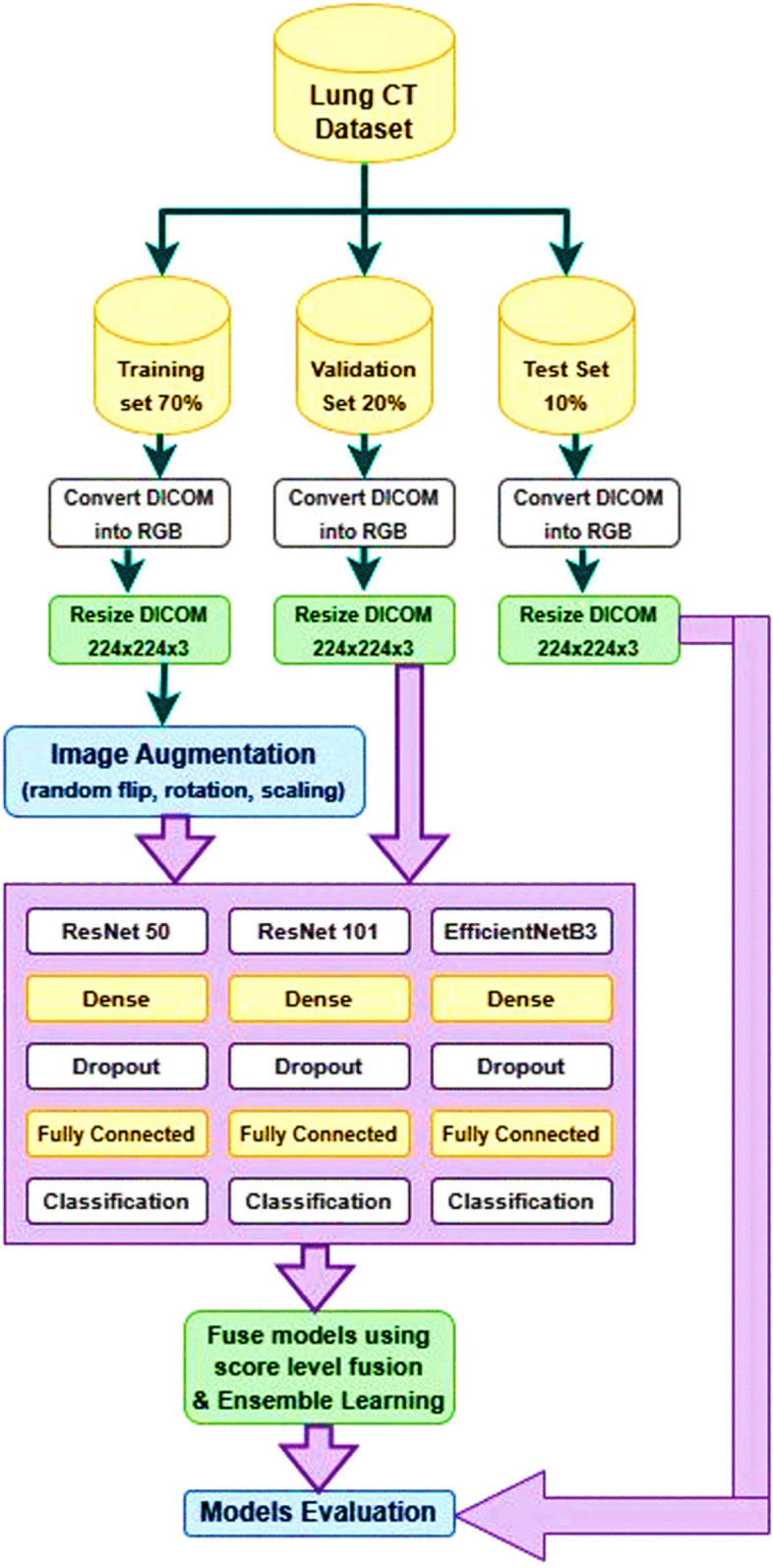
Fusion of three deep learning models for improved lung cancer diagnosis

Figure 6 presents a comprehensive diagram of the lung cancer determination strategy, highlighting the key methods included. At first, the lung CT imaging dataset is obtained. Hence, the preparation, approval, and test sets experience an arrangement of image-processing procedures to guarantee compatibility with the deep learning organized input layer. These strategies incorporate RGB change and scaling into a 224*224 arrangement.

To improve the preparation to prepare and empower the demonstration to memorize different levels of image corruption, in this manner anticipating overfitting and progressing the preparing to arrange, the preparing set is advance altered through information increase. This step includes turning, flipping, and zooming the lung CT image to create different forms of the same CT image.

Vertical flipping, zooming, and turning are utilized as modifiers for image control. The three models, specifically ResNet-50, ResNet-101, and EffecientNetB3, are at that point prepared and approved utilizing these procedures. These models were chosen based on their adequacy in image classification errands, with EffecientNet-B3, ResNet-50, and ResNet-101 being prevalent profound demonstrate sorts as shown in Table 2. EffecientNet-B3 is considered a low-computation profound demonstration. To address the lung cancer conclusion issue, the exchange learning method is utilized to retrain the same pre-trained deep learning models. This includes consolidating extra layers into the insightful plan.

The proposed deep learning models incorporates a crucial show, to be specific ResNet-50, ResNet-101, or EfficientNet-B3, taken after by a bunch normalization layer, a thick layer with 256 neurons, and 'ReLU' enactment work, a dropout layer with a 35% dropout rate, and a classification layer with a 'Softmax' enactment work and four neurons speaking to the targets. All models will be built utilizing the Adam optimizer with a learning rate of 0.01, as per the chosen preparation criteria. The connected misfortune work for this issue is the categorical cross-entropy because it could be a multi-class classification issue. The chosen execution metric is precision. The bunch estimate being utilized is 50. To decide when to end the preparing handle, a resistance level of 5 is set, meaning that if the watched degree does not move forward after 5 preparing emphasis, the method will halt. The degree being followed for this reason is the approval precision. Furthermore, the learning rate decrease figure is 0.5. The input images size 224 × 224-pixel were used by the authors to train the first convolutional layers of the ResNet model with a stride of two. Using ReLU activation functions, nonlinearity is integrated into the network design. With these designs, the images provided need to be appropriately downscaled to enable the feature extraction.

Using categorical cross-entropy as the loss function and accuracy as the selected performance indicator, the study takes use of multi-class classification. 50-person batches are trained, and the training is terminated when the validation precision does not increase above a tolerance level of five rounds. Convergence is improved during training by reducing the learning rate by a factor of 0.5.

Each of the three transfer learning models (ResNet-50, ResNet-101, and EfficientNet-B3) uses a learning rate of 0.001 using the Adam-Optimizer while training the model to classify lung cancer by analysing CT scan images. It was found through the study of learning behaviour that ResNet-50 has a saturation at epoch 32, whereas ResNet-101 and EfficientNet-B3 may also have a saturation near epoch 32, depending on their convergence speed and complexity. Observing the learning rate saturation is vital for interpreting the training dynamics of the model and refining the training strategy.

The demonstrated ResNet-50-Dense-Dropout experienced preparing with the preparing set and was assessed utilizing the assessment set. After this, the prepared show was surveyed utilizing the test set and assessment measurements. Additionally, the demonstrated ResNet-101-Dense-Dropout was prepared to utilize the preparation set and tried utilizing the assessment set. The prepared show was at that point assessed utilizing the test set and assessment measurements. The Efficient-B3-Dense-Dropout demonstration was moreover prepared to utilize the preparing set and tried utilizing the assessment set. The prepared show was at that point put to the test utilizing the test set and appraisal criteria. The three preparing models were combined at the score level, and the combined demonstration was evaluated. Also, a gathering was made utilizing the stacking outfit strategy, comprising the ResNet-50-Dense-Dropout, ResNet-101-Dense-Dropout, and Efficient-B3-Dense-Dropout models. The learned outfit demonstration was tried utilizing the test set and assessment measurements.

### Experimental analysis

**Fig. 7:**
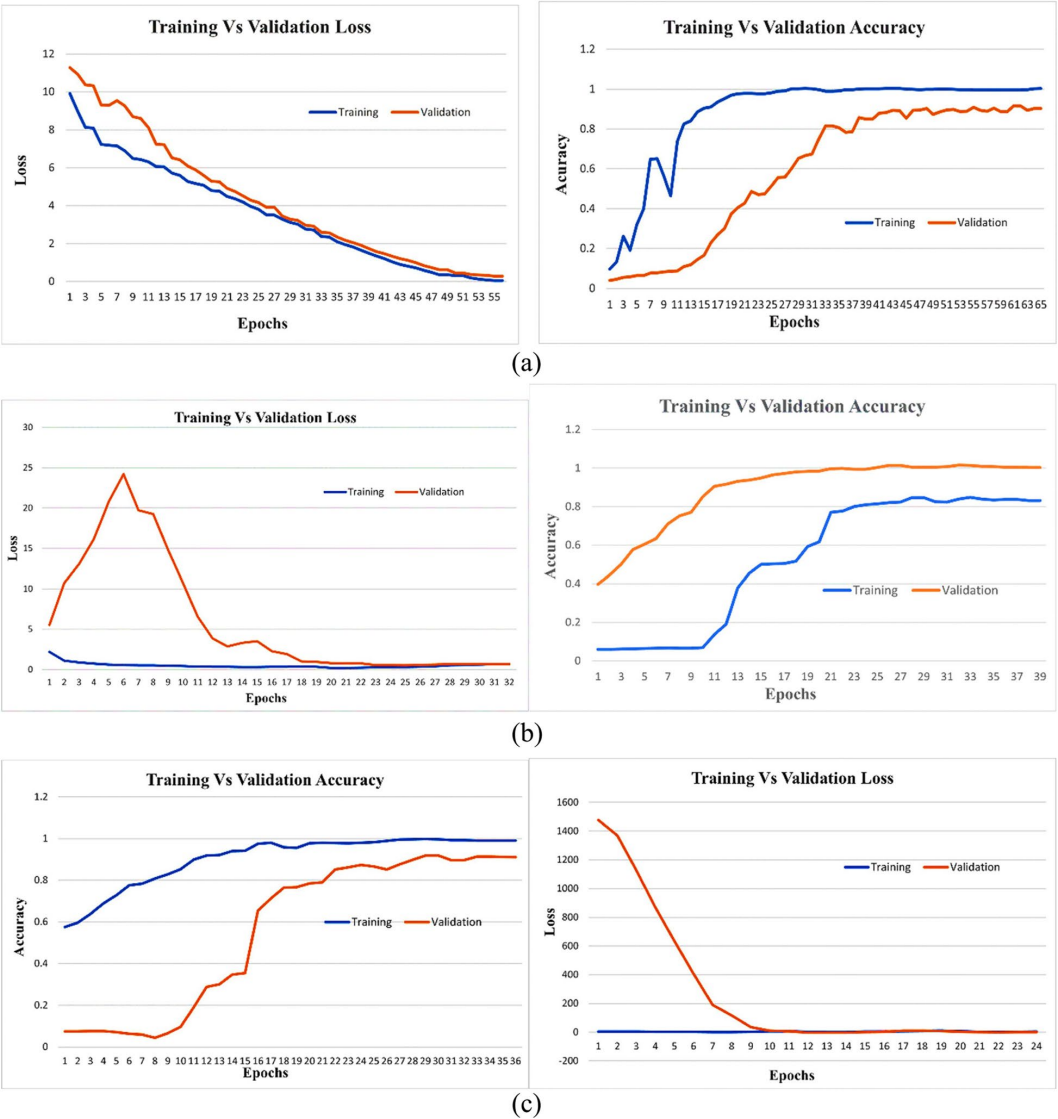
**a** EfficientNetB3-Dense Dropout: Training Vs Validation Loss and Accuracy Curves. **b** ResNet-50-Dense Dropout: Training and Validation Loss and Accuracy Curves (**c**) ResNet-101-Dense Dropout: Training and Validation Loss and Accuracy Curves

All models experience preparing to utilize the past cases. The preparing ages are utilized to calculate the exactness and misfortune for both the preparation and approval sets. Besides, the perfect approval esteem is decided for each circumstance. The exactness and misfortune bends are outwardly spoken to in Fig. 7. EfficientNet-B3 is a CNN architecture that belongs to the EfficientNet family, designed to achieve a balance between computational efficiency and model performance. It is characterized by a compound scaling method that uniformly scales the network width, depth, and resolution. Specifically, the "B3" variant represents a particular set of scaling coefficients applied to the baseline architecture, resulting in a model that is computationally efficient while maintaining competitive accuracy across various computer vision tasks. EfficientNet-B3 has been widely used in image classification, object detection, and other visual recognition tasks due to its effectiveness in achieving a favorable trade-off between model size and performance. The EfficientNet-B3 show accomplishes its best execution in terms of misfortune and exactness at ages 40 and 32, separately. On the other hand, the ResNet-50 demonstrates its ideal execution at age 15, considering both exactness and misfortune. As for the ResNet-101 demonstration, ages 14 and 15 are recognized as the ideal focuses for precision and misfortune, individually.

The EfficientNetB3-Dense model is improved by dropout layers and exhibits notable differences in training, validation loss, and accuracy curves (Fig. 7 (a)). During training, the model gradually reduces loss and increases accuracy, indicating effective learning. However, in the validation set, performance plateaus or slight fluctuations occur, indicating potential over-adjustment concerns. Fine-tuning of hyperparameters or adjustment of dropout rates could be explored to improve generalization performance. The hyperparameters taken into consideration are shown in Table 4. The layered architecture for the transfer learning model incorporating ResNet-50, ResNet-101, and EfficientNet-B3 with the specified configurations is shown in Table 5.

**Table 4:** Hypermeters consideration

Hyperparameter	ResNet-50	ResNet-101	EfficientNet-B3
Input Image Size	224 × 224 pixels	224 × 224 pixels	224 × 224 pixels
Kernel Sizes	7 × 7, 1 × 1, 3 × 3, 5 × 5	7 × 7, 1 × 1, 3 × 3, 5 × 5	NA
Stride (Initial Convolution)	2	2	NA
Stride (Subsequent Convolution)	1	1	NA
Activation Function	ReLU	ReLU	ReLU
Number of Layers	50	101	NA
Residual Blocks	Yes	Yes	NA
Global Avg Pooling	Yes	Yes	Yes
Compound Scaling	No	No	Yes
Squeeze-and-Excitation Blocks	No	No	Yes

**Table 5:** Transfer learning model incorporating ResNet-50, ResNet-101, and EfficientNet-B3 with the specified configurations

Layer (type)	Output Shape	Param #	Connected to
input_image (InputLayer)	(224, 224, 3)	0	–
resnet50_base (Functional)	(7, 7, 2048)	23,587,712	input_image[0][0]
resnet101_base (Functional)	(7, 7, 2048)	42,658,176	input_image[0][0]
efficientnetb3_base (Functional)	(7, 7, 1536)	10,783,535	input_image[0][0]
global_average_pooling2d	Global (2048)	0	resnet50_base[0][0]
global_average_pooling 2d_1	Global (2048)	0	resnet101_base[0][0]
global_average_pooling2d_2	Global (1536)	0	efficientnetb3_base[0][0]
dense_layer_1 (Dense)	(128)	262,272	global_average_pooling2d[0][0]
dense_layer_3 (Dense)	(128)	262,272	global_average_pooling2d_1[0][0]
dense_layer_5 (Dense)	(128)	196,736	global_average_pooling2d_2[0][0]
dropout_1 (Dropout)	(128)	0	dense_layer_1[0][0]
dropout_3 (Dropout)	(128)	0	dense_layer_3[0][0]
dropout_5 (Dropout)	(128)	0	dense_layer_5[0][0]
dense_layer_2 (Dense)	(64)	8256	dropout_1[0][0]
dense_layer_4 (Dense)	(64)	8256	dropout_3[0][0]
dense_layer_6 (Dense)	(64)	8256	dropout_5[0][0]
dropout_2 (Dropout)	(64)	0	dense_layer_2[0][0]
dropout_4 (Dropout)	(64)	0	dense_layer_4[0][0]
dropout_6 (Dropout)	(64)	0	dense_layer_6[0][0]
output_layer (Dense)	(4)	260	dropout_2[0][0] dropout_4[0][0] dropout_6[0][0]
output_activation (Activation)	(4)	0	output_layer[0][0] output_layer[1][0] output_layer[2][0]

The ResNet-50-Dense Dropout model shows impressive performance in terms of training and validation losses as well as accuracy curves (Fig. 7(b)). During the training phase, the model effectively minimizes losses and shows a constant decline over the years. At the same time, the accuracy of training has been consistently improved, indicating the ability of the model to learn and generalize training data.

In the validation phase, the model shows its robustness by achieving low validation losses, indicating a good generalization to invisible data. The validation accuracy curve reflects training accuracy and confirms the model's ability to perform well in new and varied samples.

The integration of dense dropouts into ResNet-50 architecture seems to contribute positively to model training dynamics, improving generalization and overall performance.

The ResNet-101 training and validation loss curves show that the model minimizes error during training and can generalize to invisible data (Fig. 7 (c)). The decrease in the trend of the two curves indicates effective learning, but the gap between them may be expanding, indicating overfitting. Table 6 provides a detailed description of the hyperparameters for each model, including training accuracy, testing accuracy, training loss, and testing accuracy.

**Table 6:** Training and testing loss vs accuracy for efficient-B3, ResNet50, and ResNet101

Model	Loss	Accuracy	Validation Loss	Validation Accuracy	F1-Score	Best Epoch	Last Epoch
ResNet50	0.01	1	0.09	0.95	0.85	23	32
ResNet101	0.02	0.99	0.12	0.95	0.84	32	35
EfficientNet-B3	0.02	0.99	0.27	0.89	0.77	31	38

The accuracy curve shows the correctness of the model in the prediction. As the accuracy of training increases, the model learns from the training data. At the same time, validation accuracy indicates the extent to which the model can be generalized to new invisible data. The combination of balanced growth in both is ideal, showing robust learning without over- or under-adaptation. It is essential to monitor convergence, divergence, or plateau signs in these curves, assess model training progress, and identify potential problems such as over-adaptation. Figure 7 delineates the preparation and approval precision and misfortune bends. Thick dropouts, such as EfficientNetB3-Dense-Dropout, ResNet-50-Dense-Dropout, and ResNet-101-Dense-Dropout, are a few of the models showcased.

**Fig. 8:**
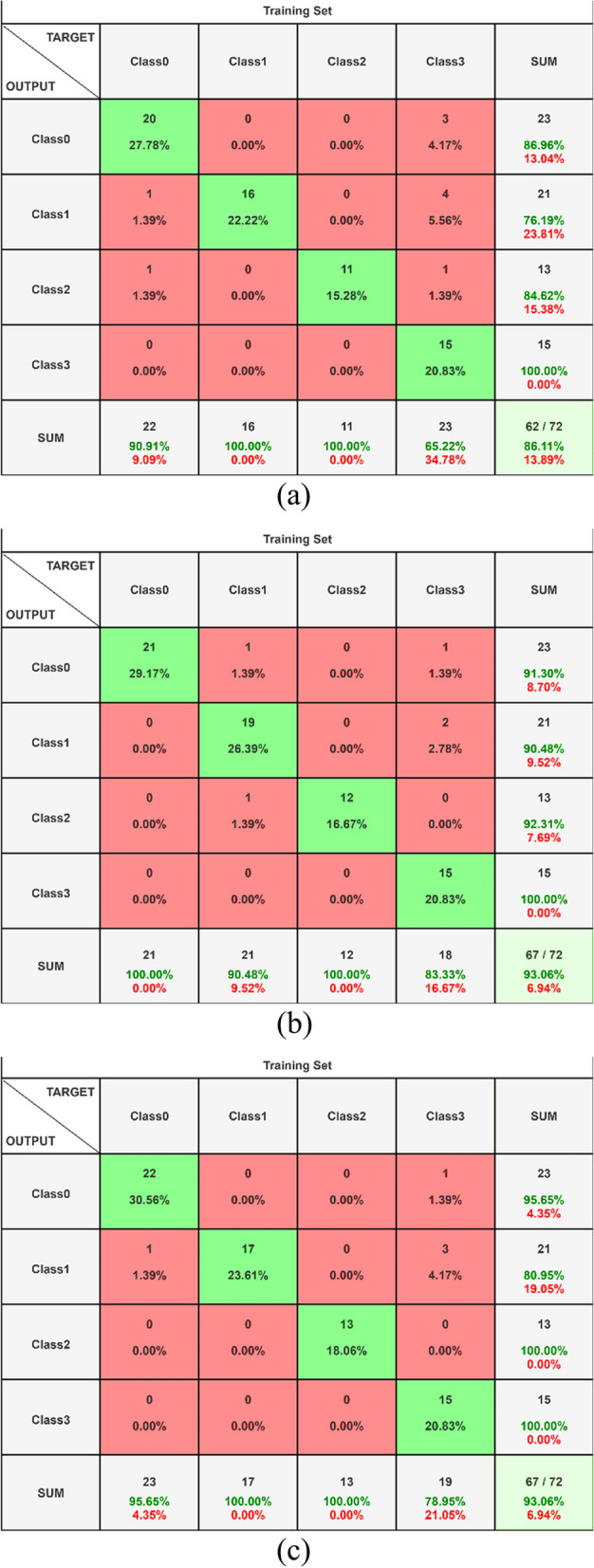
**a** Confusion Matrix EfficientNet-B3 with Dense Dropout (**b**) Confusion Matrix ResNet-50 with Dense Dropout (**c**) Confusion Matrix ResNet-101 with Dense Dropou

Class 0 refers to normal CT-image, Class 1 to Large Cell Carcinoma, Class 2 to Adenocarcinoma, and Class 3 to Squamous Cell Carcinoma in this study. EfficientNet-B3, delineated in Fig. 7, illustrates the foremost ideal merging among the models. It accomplished a test exactness of about 93.05%, an approval precision of 94.99%, and a preparing exactness of 97.5%. In differentiation, the ResNet-50 demonstration displayed preparing, approval, and test exactness scores of 97.5%, 75%, and 80.55% individually. Also, the ResNet-101 show showcased preparing, approval, and test precision scores of 100%, 94.99%, and 93.50% separately. Strikingly, the ResNet-101 demonstrates shown the most reduced preparing, approval, and test misfortune, with values of 0.0003, 0.11, and 0.47 individually. The perplexing disarray network computations for the three prepared models and the score-level combination are displayed in Figs. 8 (a) to 8 (c).

Deep learning models are used to categorize lung cancer into four classes: class 0 for normal CT scans, class 1 for large cell carcinoma, class 2 for adenocarcinoma, and class 3 for squamous cell carcinoma. False Positives (FP) are when the model incorrectly predicts absence, False Negatives (FN) are when it incorrectly predicts presence, and True Positives (TP) are when the model correctly predicts the presence of lung cancer. These cases are all considered in the confusion matrix. Hence, A True Positive (TP) is when the model accurately predicts a positive outcome indicating the presence of lung cancer and the prediction aligns with objective truth. If the model accurately predicted a negative outcome, indicating that there was no lung cancer, and the prediction was in line with the fundamental truth, this is known as a true negative (TN). False Positive (FP) depicts the conditions in which the model erroneously predicted a positive outcome indicating the existence of lung cancer, yet the prediction contradicted the essential reality. Situations when the model incorrectly predicts a negative outcome, indicating the absence of cancer, are known as false negatives (FN).

The EfficientNet-B3 outperforms all other person models in terms of results, as illustrated by Fig. 8, where the disarray matrix's primary pivot contains most of the hits. Besides, the number of wrong positives and wrong negatives is lower compared to other models. The score-level combination yields profoundly comparable results. Figure 8 outlines that both the combined and EfficientNet-B3 models show indistinguishable predominant execution, outflanking the isolated ResNet models. For a comprehensive execution comparison over all models and the four categories, refer to Table 7. In this study, class 0 normal CT-image, class 1 large cell carcinoma, class 2 adenocarcinoma, and class 3 squamous cell carcinoma are identified.

**Table 7:** Comparison of dense dropout deep learning models for cancer detection performance

Model	Precision				
	**Adenocarcinoma**	**Large-Cell**	**Normal**	**Squamous cell**	**Average**
EffiecientNetB3-Dense-Dropout	0.87	0.76	0.85	1.00	0.87
ResNet-50-Dense-Dropout	0.91	0.9	0.92	1.00	0.93
ResNet-101-Dense-Dropout	0.96	0.8	1.00	1.00	0.94
Score-level fusion model	0.92	0.9	0.92	1.00	0.94
**Model**	**Recall**				
	**Adenocarcinoma**	**Large-Cell**	**Normal**	**Squamous cell**	**Average**
EffiecientNet-B3-Dense-Dropout	0.91	1.00	1.00	0.65	0.89
ResNet-50-Dense-Dropout	1.00	0.90	1.00	0.83	0.89
ResNet-101-Dense-Dropout	0.95	1.00	1.00	0.79	0.93
Score-level fusion model	1.00	1.00	1.00	0.75	0.94
**Model**	**F1-Score**				
	**Adenocarcinoma**	**Large-Cell**	**Normal**	**Squamous cell**	**Average**
EffiecientNet-B3-Dense-Dropout	0.89	0.86	0.92	0.79	0.87
ResNet-50-Dense-Dropout	0.95	0.9	0.96	0.91	0.87
ResNet-101-Dense-Dropout	0.96	0.89	1	0.88	0.93
Score-level fusion model	0.96	0.95	0.96	0.86	0.93

Figure 8 portrayed the disarray lattice of the prepared models in the combination of ResNet and EfficientNet-B3 at the score level, alongside the EfficientNet-B3-Dense-Dropout demonstration, ResNet-50-Dense-Dropout demonstration, and ResNet-101-Dense-Dropout demonstrate, were utilized in this examination.

An average accuracy of 94% is achieved with the EfficientNet-B3-Dense-Dropout model, as Table 7 shows, indicating better performance. And even with constant accuracy and F1-score, integrating all models at the score level improves precision by 1%. Further highlights the "Normal" category's importance in obtaining the best accuracy across all classes, the "Squamous" category's highest recall, and the "Normal" category's highest F1 score. ResNet-101 further performs better than ResNet-50 in a variety of real-world circumstances.

Table 7 provides a detailed comparison of the accuracy of the main models and illustrates how effective each model is in terms of time. ResNet-50 showed that it could analyse data in 12.49 s each iteration, but ResNet-101 took a little longer 15.41 s to do the same. However, EfficientNet-B3 showed a similar processing time, with an average of 15.32 s per iteration. Thorough time computations served as the foundation for these time measures. The chart also shows that the ensemble model outperformed all other individual models, achieving an exceptional accuracy rate of 99.44%.

The results of benchmarking for cancer diagnosis using different deep learning models are displayed in Table 8. [46] discovered increased AUC values, particularly for ISIC-2016, using a hybrid CNN on ISIC datasets. To improve breast cancer classification models, [52] use CNN on both public and private data. To precisely locate and classify breast cancer, [38] use Pa-DBN-BC to histopathological images. On the LIDC-IDRI file, [27] demonstrates the precise identification of lung nodules using CNNs. Using Inception V3 on genomic datasets, [56] classify hepatocellular carcinoma with high accuracy and AUC. Utilizing a fractional backpropagation MLP, [64] was able to surpass BP-MLP in the categorization of leukaemia malignancy. An extraordinary rate of breast cancer detection was achieved by [65] by using the Modified Entropy Whale Optimization Algorithm to several datasets. Finally, better accuracy in the prediction of various forms of lung cancer is achieved in the current work by utilizing EfficientNet.

**Table 8:** Benchmarking of deep learning models for cancer detection

Study	Field description	DL model	Dataset	Results
[27]	Exact aspiratory knob discovery	Convolutional Neural Networks (CNNs)	LIDC-IDRI dataset	92.7% distribution probability with 1 bad positive per filter and 94.2% distribution probability with 2 bad positives per filter for lung nodules over 888 examinations in the LIDC-IDRI dataset. The use of MIP imaging increases the likelihood of indication and reduces the number of false positive results when locating pulmonary lymph nodes programmed into the CT interface
[39]	Pa-DBN-BC	Deep Belief Network (DBN)	The slide histopathology image dataset from four distinct cohorts achieved	86% accuracies in breast cancer location and classification, surpassing previous deep learning strategies
[56]	Hepatocellular carcinoma (HCC)	Inception V3	Genomic Data Commons Databases	96.0 accuracy for kind and dangerous classification—89.6 accuracy for tumor separation (well, direct, and destitute)—Expectation of 10 most common changed qualities in HCC—Outside AUCs for 4 qualities (CTNNB1, FMN2, TP53, ZFX4) extending from 0.71 to 0.89—Utilize of convolutional neural systems to help pathologists in classification and quality transformation discovery in liver cancer
[46]	Dermo Expert	Hybrid-CNN	ISIC-2016, ISIC-2017, ISIC-2018	AUC: 0.96, 0.95, 0.97; Improved AUC by 10.0% (ISIC-2016) and 2.0% (ISIC-2017); Outperformed by 3.0% in balanced accuracy (ISIC-2018)
[64]	Learning Algorithm for Adaptive Signal Processing	Fractional Backpropagation MLP	Leukemia cancer classification	Outperformed BP-MLP in convergence rate and test accuracy
[65]	Breast Cancer Discovery and Classification	Modified Entropy Whale Optimization Algorithm (MEWOA)	In the breast, MIAS, CBIS-DDSM	IN breast: 99.7%, MIAS: 99.8%, CBIS-DDSM: 93.8%
Current Study	Adenocarcinoma, Expansive Cell Carcinoma, Squamous Cell Carcinoma, Typical	Adenocarcinoma, expanding cell carcinoma, squamous cell carcinoma	1000 images from the Kaggle lung cancer dataset	Best accuracy for humans (EfficientNet 93%) Accuracy 99.44% synthetic accuracy

By contrast with earlier state-of-the-art methods in the Comparative Analysis of Lung Cancer Prediction using the Deep Learning technique, Table 9 demonstrates the performance and efficiency of the present advancement. The present advancement's supremacy is highlighted by this comparison. The superiority of the ensemble model over the EfficientNet-B3 and ResNet-101 models is evident, with an improvement of 6.44% and 18.44%, respectively. While the validation accuracy of the ensemble model is comparable to that of the EfficientNet-B3 and ResNet-101 models, its significant enhancement lies in achieving a precision of 99.44%.

**Table 9:** Comparative analysis of lung cancer prediction through deep learning

Aspect	Implementation Platform	Dataset Details
Platform Used	Deep learning models: ResNet-50, ResNet-101, EfficientNet-B3	LIDC-IDRI repository
Input Data	DICOM lung cancer images	1,000 images
Data Partitioning	Training: 70% Validation: 20% Testing: 10%	Training: 613 images Validation: 315 images Testing: 72 images
Model Architecture	ResNet-50, ResNet-101, EfficientNet-B3	–
Preprocessing Techniques	Data augmentation strategy	–
Classification Performance	Fusion Model: 100% precision in classifying Squamous Cells	Precision: ResNet-50, EfficientNet-B3, and ResNet-101 achieved 90%, followed by EfficientNet-B3 and ResNet-101 with slightly lower precision
Model Training	Epochs: 35 Batch Size: 32	–
Learning Rate	Adam optimizer with a learning rate of 0.001	–
Total Parameters	10,988,787	–
Trainable Parameters	10,099,090	–
Non-trainable Parameters	889,697	–
Achievements	Improved accuracy in predicting lung cancer subtypes	Potential for advancements in healthcare and reduction in mortality rates associated with lung cancer

Although there have been advancements in predicting lung cancer, it is important to acknowledge the existing limitations in the current thinking [66]. These restrictions involve using small data sets and specific scientific models [67, 68].

To isolate the region of interest (ROI) or lung tissues from lung images, it is essential to use preprocessing techniques such as image segmentation [12].

The research paper emphasizes how important it is to compare analyses with contemporary models. It focuses on the modelling architecture, learning rate, model training, implementation platform, data set details, model architecture, preprocessing techniques, classification performance, and results. Utilizing deep learning models such as ResNet-50, ResNet-101, and EfficientNet-B3, the study makes use of the LIDC-IDRI-Speicher, which has 1.000 DICOM images of lung cancers. Seventy percent of the data will be used for training, twenty percent for validation, and ten percent will be used for tests. ResNet-50, ResNet-101, EfficientNet-B3, and preprocessing data augmentation techniques are used in the model architecture. In the classification of squamous cells, the fusion model achieves 100% absolute accuracy, whereas ResNet-50, EfficientNet-B3, and ResNet-101 show 90% accuracy. The training procedure takes place across 35 epochs with a batch size of 32, using the Adam optimizer with a learning rate of 0.001. The study makes use of 10,988,787 parameters and highlights the potential for advancements in medical care as well as a reduction in mortality rates related to lung cancer through improved lung cancer subtype prediction accuracy.

The authors advocate the utilization of EfficientNet-B3 and ResNet-50–101, deep neural network algorithms, for the early detection of lung cancer. The study leverages pre-trained Convolutional Neural Networks (CNNs) and employs strategies on the LIDC DICOM datasets. All shape and texture images within the dataset are utilized for feature extraction. Notably, the automatic extraction of shape features is facilitated by the capabilities of EfficientNet-B3 and ResNet, while AlexNet is employed to prepare the highest resolution.

The research emphasizes the significance of evaluating the network input layer and the number of initial layers to enhance the efficiency and accuracy of the proposed system. Furthermore, the article highlights the successful completion of all training procedures, including lung separation and elimination processes. The system's performance is rigorously assessed, achieving 100% in sensitivity, precision, and accuracy, with low false rates. The study underscores the importance of further analysis, particularly in methods like segmentation, which may necessitate a comprehensive evaluation of the entire image dataset.

The proposed diagnostic approach holds promise in providing elite medical professionals with precise and timely diagnostic impressions. The robust performance metrics and successful completion of various procedures underscore the potential for the proposed system to contribute significantly to early lung cancer detection, paving the way for enhanced medical diagnoses in the future.

### Conclusion and future scope

In conclusion, this study examined the use of deep learning models for precise lung cancer diagnosis and classification, including ResNet-50, ResNet-101, and EfficientNet-B3. Extensive analysis of experimental data and cross-validation with prior research demonstrated the efficacy of the proposed Fusion Model, particularly in accurately diagnosing Squamous Cell Carcinoma. The remarkable 92% increase in prediction accuracy of the combined model demonstrates how revolutionary it may be for the identification and management of lung cancer. These findings highlight the potential of deep learning algorithms to offer tailored treatment regimens and ultimately reduce the mortality rate from lung cancer. To enhance patient outcomes and advance medical imaging capabilities, forthcoming endeavours ought to concentrate on refining model architectures, broadening datasets, and encouraging multidisciplinary partnerships. In the future, deep learning models can be used in a wide range of research projects and using larger datasets. Additionally, it was noted that obtaining knowledge and achieving certain scores was connected to improving health and lowering lung cancer death rates by dealing with the problem of inaccurate precision.

## Data Availability

Data may be available upon reasonable request from the corresponding author.
